# Self-aliquoting micro-grooves in combination with laser ablation-ICP-mass spectrometry for the analysis of challenging liquids: quantification of lead in whole blood

**DOI:** 10.1007/s00216-016-9717-3

**Published:** 2016-06-30

**Authors:** Winfried Nischkauer, Frank Vanhaecke, Andreas Limbeck

**Affiliations:** Institute of Chemical Technologies and Analytics, TU Wien, Getreidemarkt 9/164-IAC, 1060 Vienna, Austria; Department of Analytical Chemistry, Ghent University, Krijgslaan 281-S12, 9000 Ghent, Belgium

**Keywords:** Laser ablation-ICP-MS, Whole blood analysis, Lead quantification, Dried-droplet laser ablation, Transient signals

## Abstract

We present a technique for the fast screening of the lead concentration in whole blood samples using laser ablation-inductively coupled plasma-mass spectrometry (LA-ICP-MS). The whole blood sample is deposited on a polymeric surface and wiped across a set of micro-grooves previously engraved into the surface. The engraving of the micro-grooves was accomplished with the same laser system used for LA-ICP-MS analysis. In each groove, a part of the liquid blood is trapped, and thus, the sample is divided into sub-aliquots. These aliquots dry quasi instantly and are then investigated by means of LA-ICP-MS. For quantification, external calibration against aqueous standard solutions was relied on, with iron as an internal standard to account for varying volumes of the sample aliquots. The ^208^Pb/^57^Fe nuclide ratio used for quantification was obtained via a data treatment protocol so far only used in the context of isotope ratio determination involving transient signals. The method presented here was shown to provide reliable results for Recipe ClinChek® Whole Blood Control levels I–III (nos. 8840–8842), with a repeatability of typically 3 % relative standard deviation (*n* = 6, for Pb at 442 μg L^−1^). Spiked and non-spiked real whole blood was analysed as well, and the results were compared with those obtained via dilution and sectorfield ICP-MS. A good agreement between both methods was observed. The detection limit (3 s) for lead in whole blood was established to be 10 μg L^−1^ for the laser ablation method presented here.

**Electronic supplementary material:**

The online version of this article (doi:10.1007/s00216-016-9717-3) contains supplementary material, which is available to authorized users.

**Graphical Abstract:**
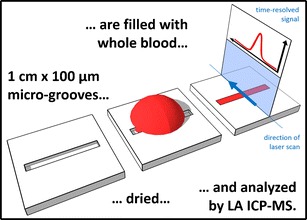
Micro-grooves are filled with whole blood, dried, and analyzed by laser ablation ICP-mass spectrometry. Notice that the laser moves in perpendicular direction with regard to the micro-grooves

## Introduction

The metal lead is known for its toxicity, both acute and chronic. By substituting tetra-ethyl lead with purely organic compounds as anti-knocking agents in petrol, by exchanging water pipes made from lead with pipes made from polymeric material and by replacing the once widely used white pigment lead white 2PbCO_3_·Pb(OH)_2_ with TiO_2_, the population is less exposed to this metal than in the past. However, in certain work-related contexts, exposure can still be high. According to a directive of the council of the European Union [1], “medical surveillance is carried out if […] a blood-lead level greater than 400 μg Pb L^−1^ blood is measured in individual workers.” To determine the lead concentration in whole blood of potentially exposed workers, suitable analytical techniques are therefore required.

Conventional approaches for analysis of lead in whole blood usually require dilution or even digestion of the blood matrix prior to measurement. After digestion by means of concentrated mineral acids and oxidizers, the sample is also diluted. This clear solution is then suitable for analysis via graphite furnace-atomic absorption spectrometry (GF-AAS) or inductively coupled plasma-mass spectrometry (ICP-MS). For digested and diluted samples, quantification can often be accomplished via external aqueous calibration or matrix-adjusted calibration. However, chemical digestion of the blood matrix comes at the cost of low sample throughput, as well as high reagent consumption, which in turn can lead to increased blank values and costs. When simply diluting the sample, matrix effects have to be taken into consideration by using standard addition. Moreover, coagulation of the blood has to be prevented, by diluting the blood as soon as possible after collection.

Alternative approaches that avoid mineralization or dilution of blood can be found in the field of solid-sampling techniques. Among others, Resano et al. have shown that it is possible to apply blood droplets onto filter paper, to cut out the dry spot obtained and to analyse it via GF-AAS for its lead content [2]. Instead of AAS, which is essentially a single-element method, Cizdziel [3] proposed to use laser ablation (LA)-ICP-time-of-flight MS for analysis of blood dried on filter paper.

However, and in contrast to solid-sampling AAS, an accurate and quantitative assessment of dried blood spots by means of LA is a challenging task. The diameter of the laser beam is in the micrometre range, whereas the diameter of a dried blood spot is typically in the millimetre range. Therefore, only a part of the sample is consumed during single-shot or spot-drilling analysis. For obtaining correct quantitative results, this small part of the sample used for analysis has to be representative of the liquid blood sample before deposition. When depositing liquids on filter paper, chromatographic splitting into different sample fractions is possible and thus might compromise the sample’s homogeneity. As such effects can vary from sample to sample, or between samples and standards, correct quantification is severely hampered. An extended discussion of the problems related with dried-droplet LA can be found elsewhere [4].

One way to solve the problem of chromatographic separation is to ablate the entire dried blood spot. Any lateral inhomogeneity is thus obviated. However, with commercial LA systems operating with repetition rates in the hertz range, ablation of such large areas can take a very long time. There are femtosecond LA systems with repetition rates in the kilohertz range and with scan speeds of several millimetres per second. Such systems can ablate one entire dried blood spot within reasonable time [5], yet they are to date not available as routine instruments. Also, recently available ultra-fast cells allow higher laser repetition rates (up to several hundred Hz) using the more traditional nanosecond LA systems [6].

Another possibility to solve the problem of non-representative sub-samples is to apply a radial scanning approach. In a recent publication, we discussed this in more detail [7]. The idea behind this concept is that any chromatographic separation of a deposited sample droplet should always be symmetrical to the centre of the dried spot. If the laser is scanned from one side to the other across the dried spot, passing through the centre of the spot, all possible chromatographic variations are recorded. However, it is necessary to produce strictly circular droplets, and the shortcoming of this approach is that sample application requires a skilled operator.

To compensate for chromatographic effects by ablating the entire droplet, while maintaining reasonable sample throughput and straightforward sample preparation, the size of the blood spot has therefore to be reduced [8]. However, pipetting volumes in the nanolitre range using pipettes or syringes is a challenging task, and localizing such small dried droplets with the microscope of the LA system is difficult. Therefore, a straightforward way for producing nanolitre sub-samples of a liquid blood sample was developed in this work.

Splitting a sample droplet into many identical sub-samples in the nanolitre range can be achieved by means of self-aliquoting micro-array plates, as proposed by Pabst et al. in the context of matrix-assisted laser desorption ionization mass spectrometry (MALDI-MS) [9]. Such self-aliquoting arrays consist of a smooth polymeric slide, which contains small cavities of several micrometres in diameter. Such cavities can be readily produced with a LA system. If a liquid droplet is swiped over such cavities, small amounts of the liquid are trapped in each cavity.

In this contribution, the initial concept described in Pabst et al. [9] was successfully transferred from MALDI-MS to LA-ICP-MS and further optimized to allow for more straightforward sample application and analysis. This approach was combined with a data treatment scheme so far only used in the context of isotope ratio determination with transient signals. The method developed was applied to the determination of lead in Recipe ClinChek® whole blood reference material and a freshly collected and spiked whole blood sample. External calibration against aqueous standard solutions and iron as an internal standard was possible.

## Materials and methods

### Reagents and standard solutions

For standard dilution, water with a resistivity of 18 MΩ cm obtained from an Easypure system (Thermo, Germany) was used throughout. Single-element standard solutions at 1000 mg L^−1^ were obtained from Merck, Germany. Four standard solutions were prepared from those stock solutions, with final lead concentrations of 26, 76, 316, and 614 ng mL^−1^. Each of these solutions contained iron at a concentration of 376 μg mL^−1^.

Recipe ClinChek® Whole Blood Control levels I–III (nos. 8840, 8841 and 8842, containing 59.1, 228 and 446 ng mL^−1^ lead as well as 379, 380 and 377 μg mL^−1^ iron, respectively) were reconstituted using Easypure water according to the manufacturer’s instructions. Reconstituted blood reference material was used immediately for analysis and not stored longer than 24 h.

A fresh whole blood sample was investigated as well. As the concentration of lead in the sample was relatively low, two levels of lead were spiked to the sample. First, the sample was split into three aliquots of 1 mL, and to each aliquot, 0.1 mL of 1 % HNO_3_ containing adequate concentrations of lead was added. The concentrations of the spike were chosen such that the resulting whole blood contained spiked concentrations of 0, 150, and 250 ng mL^−1^. The spiked volume was equal to 10 % of the initial sample volume, and should result only in minimal changes of sample matrix, compared to the original, non-spiked blood.

### Instrumental

The LA system used for production of the micro-array plates and the micro-grooves was a New Wave Research frequency-quintupled Nd:YAG solid-state laser operating at 213 nm. The same system was used for sample analysis via LA-ICP-MS. Ablation was carried out under helium atmosphere (0.8 L min^−1^). After the ablation chamber, argon was admixed to the helium stream as make-up gas at a flow rate of 0.8 L min^−1^.

The dry aerosol produced upon laser ablation was transported into a Thermo iCAP Qc quadrupole ICP-MS unit operating under standard conditions (1550 W plasma power, nickel cones, cool gas at 14 L min^−1^, auxiliary gas at 0.8 L min^−1^, 5 ms dwell time, monitoring of the ^57^Fe and ^208^Pb ion signals, total cycle time 13 ms). Data collection was accomplished using the instrument software (QTegra^TM^) in time-resolved mode.

For comparing the laser approach presented here with a traditional nebulizer-based method, spiked and non-spiked real whole blood samples were diluted and analysed using standard addition and a Thermo Element XR sectorfield instrument. Details on this method can be found in the Electronic Supplementary Material (ESM).

### Micro-array plates and linear grooves

Two different types of arrays were investigated for blood deposition. First, micro-cavity arrays as described in Pabst et al. [9] were prepared by ablating circular craters into poly (methyl methacrylate) (PMMA) microscope slides (2.5 × 7.2 cm, Betzold, Austria) using the NWR 213 LA system. The laser parameters used for production of micro-array plates were 7.5 J cm^−2^ laser fluence, 20 Hz repetition rate, 4 s dwell time and 100 μm beam diameter. The distance between craters was 300 μm and the craters were arranged in a 4 × 4 pattern.

**Fig. 1:**
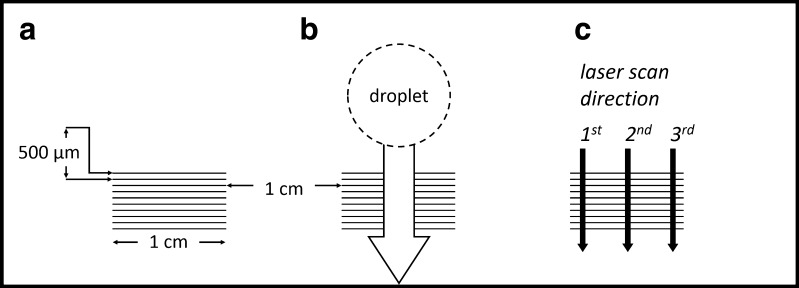
**a**–**c** Schematic of the proposed LA-ICP-MS method using micro-grooves. Each groove is 1 cm long and 100 μm wide. For analysis, the laser is scanned according to a line perpendicular to the grooves

The second, newly developed design consists of three sets of ten parallel grooves. Each of the grooves was 100 μm wide and 1 cm long (see Fig. 1a). The laser parameters used for production of the micro-grooves were 10 J cm^−2^ laser fluence, 20 Hz repetition rate, 100 μm spot diameter and 100 μm s^−1^ scan speed. To fabricate one set of ten micro grooves, roughly 15 min is required. This, and the fact that the slides are not expensive, allows for single use of the slides.

### Deposition of blood and analysis of dry residues

One small droplet (approximately 5 μL) of blood, reference material or aqueous standard solution was deposited on the polymeric slide, just next to one set of linear micro-grooves. The liquid was then spread across the slide using a rubber spatula. After passing the micro-grooves, the remaining solution was removed from the microscopic slide in one continuous motion (see Fig. 1b). Due to the small liquid volume trapped in each groove or micro-array, the samples dried quasi instantly. This approach was used for both types of slides containing micro-cavities following the design of Pabst et al. [9] as well as for the newly developed design using micro-grooves.

The dried samples were analysed by means of LA-ICP-MS. Laser parameters used were 3.1 J cm^−2^ laser fluence, 20 Hz repetition rate, 200 μm spot diameter and 100 μm s^−1^ scan speed. In the case of the micro-cavities following the design by Pabst et al. [9], the laser traces used for analysis were adjusted such that each cavity was consecutively ablated. In the case of the newly designed micro-grooves, the LA pattern was arranged in a way that each line scan crosses the grooves perpendicularly (see Fig. 1c).

## Results

### Optimizing the design of the cavities

In a first approach, micro-array plates with circular cavities were prepared and filled with aqueous standard solutions which were dried and analysed by LA-ICP-MS. The design of the cavities corresponds to the one described in [9] with minor variations. When analysing an aqueous standard with 400 ng mL^−1^ lead, typical values for the relative standard deviation were around 5 % (*n* = 16, 16 individual integrated peak areas, one for each cavity, using iron as an internal standard to compensate for varying filling of individual cavities).

However, when filling the circular micro-cavities with whole blood reference material, the results obtained were not satisfying. Firstly, when wiping the blood swiftly over the cavities, also the area between the cavities was found to produce significant signals upon LA-ICP-MS analysis. To circumvent this problem, the rubber spatula was wiped over the cavities applying higher pressure. This in turn resulted in very low peak areas, since a large part of the blood is removed again from the cavities during the swiping step.

To overcome this problem, the design of the micro-cavities was changed from circular cavities to long micro-grooves. Such grooves have the advantage that they are filled with a sufficient quantity of blood while having only negligible contamination in between the individual grooves. Additionally, it is possible to see the micro-grooves with the naked eye, facilitating sample deposition. Figure 1 shows the final design of the optimized micro-groove cavities. To the best of the authors’ knowledge, this is the first time such a design is proposed for quantitative analysis of liquid samples.

### Figures of merit and evaluation of internal standard

Four sets of the newly designed micro-groove cavities were filled with aqueous standard solutions and a blank solution (containing only iron), and analysed by LA-ICP-MS. The obtained transient signals for ^208^Pb and ^57^Fe were integrated (left boundary: first steep increase of the iron signal, right boundary: lead signal drops back to instrumental background level) and the ratio ^208^Pb/^57^Fe was calculated. From the standard deviation of six blanks, the detection limit was found to be 10 μg L^−1^ lead (3 s).

The repeatability of the lead signal at a concentration of 600 ng mL^−1^ was found to be approximately 5 % for *n* = 3 repetitions from one set of micro-grooves. The linearity of the calibration curve improved upon using iron as an internal standard, indicating that slightly different volumes of sample are trapped in each set of micro-grooves. As explained in [9], the amount of liquid trapped within one circular micro-cavity depends on the depth of the cavity and the surface structure and roughness at the edge of the cavity. Also, the amount of liquid trapped within a cavity depends on the liquid’s properties. Therefore, a difference in matrix can lead to a difference in sample volume retained in each cavity, making it necessary to use iron as an internal standard.

### Data treatment

Integration of transient signals is a commonly applied way for data treatment in laser ablation and was also used here to determine the detection limit. However, integration requires manual setting of integration boundaries, and all data (background and actual signal) contribute equally to the result [10, 11]. An alternative data treatment approach for obtaining isotope ratios of transient signals was developed by Fietzke et al. [10, 11]. In the past, this approach has been used in the context of multi-collector ICP-MS, also in combination with LA, but to the best of our knowledge, this is the first time it was evaluated using LA single-collector ICP-MS.

**Fig. 2:**
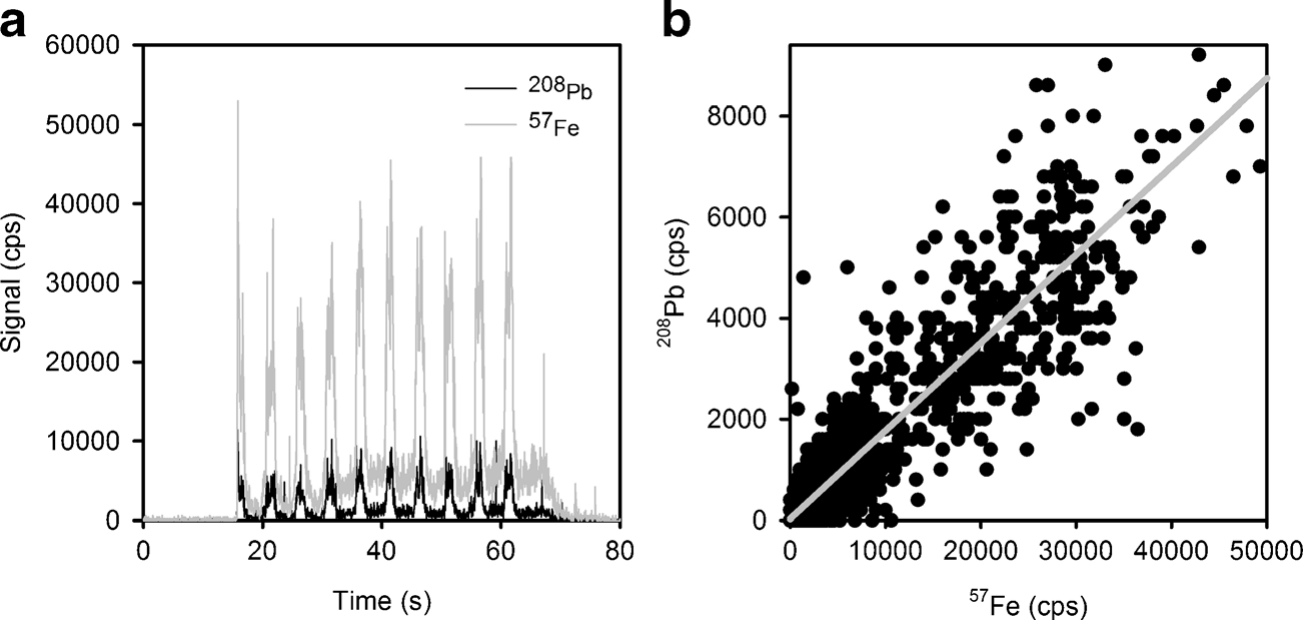
Example of data treatment. The transient signals are recorded (**a**); the entire data of one such scan are then plotted in a Pb/Fe plot (**b**); the slope of the interpolated straight line corresponds to the Pb/Fe ratio

For each sample, all data points recorded in one measurement (i.e. ten transient peaks including gas blank in between; see Fig. 2a) were plotted in a ^208^Pb/^57^Fe plot. Then, the best-fitting straight line was traced through the data points, its slope representing the ^208^Pb/^57^Fe ratio (see Fig. 2b). Dronov and Schram [12] have recently used the described method in combination with single-collector quadrupole ICP-MS and liquid samples. The authors suggested to use orthogonal distance regression (ODR) instead of conventional least-squares regression [12], to account for the fact that values on the abscissa as well as values on the ordinate are influenced by uncertainties [13]. Therefore, the ODR package of OriginPro 2016G was used for data treatment throughout this work.

The advantage of the presented type of data treatment is that data points with a high signal intensity obtain more statistical weight than do data points close to the instrumental background, which is due to the leverage effect. Hence, the blank values basically do not contribute to the slope of the curve, and the entire dataset can be used directly, obviating the necessity to manually set integration boundaries. This allows for a very straightforward data treatment protocol. However, as the lead concentration decreases, the slope does not reach zero, but rather the correlation coefficient of the interpolated line deteriorates. This means that with this method it is not possible to determine detection limits in the conventional way. However, if there is significant signal on both isotopes, this method allows for a very straightforward quantitative data evaluation.

### Analysis of whole blood samples

For analysis, the aqueous standard solutions, the three reference material levels, as well as the three levels of spiked real whole blood were applied onto micro-grooves. LA-ICP-MS analysis of the samples and standards was performed, resulting in a set of transient signals similar to the one depicted in Fig. 2a. The time-resolved ICP-MS signal thus consisted of ten transient lead and iron signals, each one a result of passing the laser beam over each of the ten micro-grooves in perpendicular direction. Slight variations in signal height are due to differences in sample volume trapped in each micro-groove. Since the volume of sample trapped within the micro-grooves is not known, iron was used as an internal standard. This also compensated for different retention efficiencies in the micro-grooves due to different matrix compositions (differences in between blood samples or in between blood and aqueous standards). The ^208^Pb/^57^Fe ratio was calculated from the slope of an interpolated straight line, as described above. All samples were quantified using external, aqueous standards.

With the method developed, Recipe ClinChek® whole blood control sample levels I, II and III (order nos. 8840, 8841 and 8842, respectively) were analysed for their lead concentration. For quantification, aqueous standards with Fe and Pb concentrations close to the reference material were used. For each sample or standard, two sets of micro-grooves were analysed three times each. One line scan was performed in the centre of the micro-grooves; the other two were performed on each side (see Fig. 1c). There was no significant difference in terms of Pb/Fe ratio between the three positions. The results were found to be in very good agreement with the certified values: level I (reference value 59.1 ± 11.8 ng mL^−1^, found 58 ± 12 ng mL^−1^), level II (reference value 228 ± 46 ng mL^−1^, found 228 ± 6 ng mL^−1^) and level III (reference value 446 ± 89 ng mL^−1^, found 442 ± 10 ng mL^−1^; all data: *n* = 6).

To further investigate the capabilities of the method, a fresh whole blood sample was analysed, which was spiked with two increasing concentrations of lead. From each of these real whole blood samples, one aliquot was analysed by the abovementioned laser ablation method. The samples were also analysed by conventional ICP-MS analysis, by diluting the blood 100-fold and performing standard addition quantification using indium as an internal standard. Details regarding this conventional method can be found in the ESM. Both methods are in good agreement with regard to the found lead concentration (LA method 137 ± 10 and 286 ± 22 μg L^−1^, dilution method 135 ± 8 and 240 ± 17 μg L^−1^, for level 1 and level 2, respectively). However, in the non-spiked whole blood sample (level 0), concentrations were below the detection limit of the LA method.

## Discussion

It has to be considered that the iron concentration in the whole blood reference material is known and constant in all three levels of the certified reference material. This makes internal standardization very straightforward. In the real sample, the iron concentration was approximately 20 % higher than in the reference material and in the aqueous standards. This could lead to an underestimation of the lead concentration in the present case. Nevertheless, it was possible to distinguish between different levels of lead concentration and to obtain a reasonable agreement with the reference method.

In the future, when analysing a wide range of real blood samples, natural variation of the iron concentration is expected and has to be taken into consideration. For healthy individuals, the concentration of iron in whole blood lies typically around 450 mg L^−1^ (309–521 mg L^−1^ [14], 425–500 mg L^−1^ [15], 445–521 mg L^−1^ [16]). Variation in the iron concentration can therefore also affect the quantification of lead. However, the aim of the method presented here is to spot samples with anomalously high lead concentrations (400 μg L^−1^ or higher, as compared to the typical lead concentration of 40 μg L^−1^ [17] in the non-occupationally exposed population). Variations in iron concentration should therefore not lead to false-negative results for the lead concentration. In other words, even with natural variations in the iron concentration, anomalously high lead concentrations will still be detected. Samples thus identified can then be investigated with more accurate but more time-consuming conventional methods. However, monitoring the iron level in whole blood would improve the accuracy of the analysis if needed in a different context. Along the same lines, accuracy can also be further improved by monitoring all four, or at least the three most abundant, isotopes of lead to take into account natural variation in the isotopic composition of the element.

## Electronic supplementary material

Below is the link to the electronic supplementary material.

ESM 1 (PDF 250 kb)
