# Supplementary material for: Self-aliquoting micro-grooves in combination with laser ablation-ICP-mass spectrometry for the analysis of challenging liquids: quantification of lead in whole blood
Source: Anal Bioanal Chem. 2016 Jun 30;408:5671–6. doi: 10.1007/s00216-016-9717-3 (PMC4958394; doi:10.1007/s00216-016-9717-3)
Supplement: Supplementary file 1 — (PDF 250 kb) [file 216_2016_9717_MOESM1_ESM.pdf]

Analytical and Bioanalytical Chemistry

Electronic Supplementary Material

Self-aliquoting micro-grooves in combination with laser ablation-ICP-mass spectrometry for the analysis of challenging liquids: quantification of lead in whole blood

Winfried Nischkauer, Frank Vanhaecke, Andreas Limbeck

Quantification of the lead concentration in spiked whole blood samples using conventional nebulizer-based ICP-MS.

1. Sample preparation

Whole blood samples (either native, or spiked with increasing amounts of lead) were diluted 100 times using 1% v/v nitric acid. All dilutions were done gravimetrically. The samples were homogenized thoroughly, and each sample was divided in five sub-samples which were spiked with adequate amounts of lead to allow for standard addition quantification. At this stage, indium was added as internal standard to all samples with a final concentration of 2 ng mL^{-1}. Samples were analysed immediately after dilution. Recipe ClinChek® Whole Blood Control level II was reconstituted freshly, and treated as unknown sample using the same dilution and standard addition conditions.

2. Instrumental

Measurements were performed on an Element XR sectorfield ICP-MS (Thermo) using nickel cones and a quartz concentric nebulizer connected to a quartz cyclonic spray chamber. The instrument was operated in low resolution mode, and tuned for best sensitivity (Li, In, U) and low oxide rates (UO/U). Further instrumental details can be found in table S1.

Table S1 Instrumental parameters of the Element XR instrument

RF power	1200	W
Cooling gas	15	L min^{-1} Ar
Auxiliary gas	0.8	L min^{-1} Ar
Nebulizer gas	1.03	L min^{-1} Ar
Sample uptake	0.3	mL min^{-1}
Scan-mode	E-Scan	
Resolution	Low	
Mass Window	100	
Samples per peak	10	
Runs/ Passes	4/5	
Detector mode	Counting	
Monitored isotopes	^{206}Pb ^{207}Pb ^{208}Pb ^{115}In	

3. Analysis

Samples were analysed in the order of increasing lead concentration. In-between each individual sample, the system was rinsed for 1 minute with 5% v/v nitric acid. After each full block of samples (i.e., all standard addition solutions corresponding to one blood sample), the system was rinsed for 5 minutes with a solution containing 5% v/v nitric acid and 0.5% v/v isopropanol. Aqueous control standards containing lead and indium as well as blank solutions were analysed periodically to monitor the performance of the instrument. No significant trend of the internal standard or the control standards over the entire measurement session was observed. Therefore, no normalization to the internal standard was carried out and signals of ^{208}Pb were directly used for quantification. The method detection limit was calculated from aqueous standard solutions and was found to be 0.6 ng g^{-1} in the undiluted whole blood.

4. Results

The correlation coefficient of the derived standard addition curves was > 0. 9 in all cases, and therefore the data was quantified using the approach described by Meija et al. (doi: 10.1021/ac5014749) which uses coordinate swapping to allow for a straight-forward assessment of the uncertainty of the calculated result obtained from standard addition.

For the Recipe ClinChek® Whole Blood Control level II, a good agreement with the certified value was found (certified value: 228 ng g^{-1} with acceptable values between 182 ng g^{-1} – 274 ng g^{-1}). All calculated data can be found in table S2.

Table S2 Results calculated using the standard addition approach

	Pb concentration (ng g^{-1})
Recipe CleanCheck®	209 ± 12
Real whole blood level 0	16 ± 1
Real whole blood level 1	135 ± 8
Real whole blood level 2	240 ± 17

The iron concentration in the whole blood sample was compared with the iron concentration in the Recipe CleanCheck® reference material. To this end, both samples were diluted 100 times, spiked with indium, and analysed in medium resolution mode using ^{57}Fe and ^{115}In in medium mass resolution using the detector in analog mode. Other instrumental conditions were similar to the ones summarized in table S1. The iron found in the whole blood amounts to 470 ± 60 mg L^{-1}.
